# Erratum

**DOI:** 10.4269/ajtmh.964err

**Published:** 2017-04-05

In *Am J Trop Med Hyg 95:* 1031–1036 by Cabada and others (https://doi.org/10.4269/ajtmh.16-0237), there was an error in the author list. Clinton A. White Jr. should have been listed as A. Clinton White Jr. The journal regrets the error.

